# USING HIGH-FREQUENCY ASSESSMENTS TO EXPLORE WITHIN-PERSON COUPLING OF PSYCHOLOGICAL STATES AND COGNITION

**DOI:** 10.1093/geroni/igad104.2045

**Published:** 2023-12-21

**Authors:** John Felt, Riki Slayday, Stacey Scott, Martin Sliwinski, Karra Harrington

**Affiliations:** Center for Healthy Aging, Penn State, University Park, Pennsylvania, United States; The Pennsylvania State University, State College, Pennsylvania, United States; Stony Brook University, Stony Brook, New York, United States; Pennsylvania State University, State College, Pennsylvania, United States; The Pennsylvania State University, University Park, Pennsylvania, United States

## Abstract

From the perspective of traditional assessment approaches, variation in cognitive performance due to participant factors (e.g. mood, fatigue) are treated as part of measurement error. This talk will discuss how high-frequency assessment methods, such as ecological momentary assessment (EMA), can be leveraged to capture meaningful signal from variation in cognitive performance. This approach will be used to explore fluctuations in thought valence and levels of arousal as short-term mechanisms for change in working memory and processing speed performance. Participants (n = 265, aged 25-65, 64.9% female) completed a 14-day measurement burst that included five EMAs per day. Each EMA included self-report measures of current thought valence (pleasant-unpleasant) and level of arousal (sleepy/tired-alert/active), as well as two brief cognitive tasks. We use multi-level modelling to disaggregate between- and within-person effects of thought valence and arousal, and to examine within-person coupling with performance on each of the cognitive tasks. Models also include demographic and contextual covariates (e.g. current location), as well as linear and quadratic functions of time and session number to account for time of day and retest effects. We found that at moments when individuals were experiencing more unpleasant thoughts than usual for them, they also made more errors on the working memory task. Additionally, at moments where individuals were feeling more sleepy/tired than usual for them, they performed more slowly on the processing speed task. These results will be discussed from the perspective of using models of within-person coupling to understand short-term mechanisms for cognitive change.

